# Ureteral stents should be soaked for several minutes before placement

**DOI:** 10.1186/s40064-015-1034-3

**Published:** 2015-06-09

**Authors:** Norbert Laube, Chintan Desai, Falk Bernsmann, Christian Fisang

**Affiliations:** Deutsches Harnsteinzentrum, Urologisches Zentrum Bonn Friedensplatz, Friedensplatz 16, 53111 Bonn, Germany; NTTF Coatings GmbH, Maarweg 32, 53619 Rheinbreitbach, Germany; Klinik für Urologie und Kinderurologie, Universitätsklinikum Bonn, Sigmund-Freud-Straße 25, 53127 Bonn, Germany

**Keywords:** DJ-stent, Friction, Hydrophilicity, Moistening, Surface free energy, Wettability

## Abstract

**Purpose:**

Placement of ureteral stents (DJ-stents) may lead to complications. Inappropriate friction properties of the implant are, inter alia, made responsible for primary injuries, injury-related inflammation and a cascade of consecutive side effects. Hydrophilicity is considered to be related to low friction. The question arises, whether the various products on the market show their respective maximum hydrophilicity directly after unwrapping or a pre-use moistening, as already routinely done with the guide wire, is necessary.

**Methods:**

The surface wettability of commercial and experimental DJ-stents was determined by water contact angle (WCA) measurements using the sessile drop method. One reference surface and 11 different stent surface types were tested. In order to determine the influence of moistening on the stents’ surface wettability, WCAs were measured twice, with dry, and soaked (30 min, 0.9%-NaCl) specimens. Each sample of a surface type was tested at three different positions to avoid effects of surface heterogeneities. Up to six samples of the same surface type were examined.

**Results:**

Mean WCAs on fresh and soaked stent surfaces ranged from 75°–103° and 71°–99°. In every case the WCAs on soaked surfaces were lower. On average the WCAs decrease by 7%, the individual decreases differ considerably, from 2 to 16%. For 7/12 of the examined surface types, the decrease in contact angle is statistically significant with *p* ≤ 0.01.

**Conclusions:**

DJ-stents freshly unwrapped show less hydrophilic properties compared to DJ-stents soaked in saline. To obtain maximum hydrophilicity at stent placement, DJ-stents should be soaked. The results may advocate a similar approach for other urological equipment.

## Background

Placement and indwelling of double-J (DJ) ureteral stents are associated with a DJ-inflicted morbidity rate of up to 80% ranging from generalized urinary tract “discomfort”, urgency or flank pain to urinary tract infection, formation of crystalline bacterial biofilm triggering obstruction and subsequent stent failure (Joshi et al. 2003; Miyaoka and Monga 2009). Much of the morbidity is related to the biocompatibility of the materials used.

Surface chemistry is of key importance in biological signaling cascades. One of the surface properties defining the interface of implant and biological environment is its wetting behavior. Investigations on biofilm- and mineralization-determining surface properties have identified hydrophilic/hydrophobic interactions as one of the cornerstones of cell-biomaterial interaction (Juliano et al. 1993; Grinnel and Feld 1982), protein adsorption (Andrade and Hlady 1986; Norde and Lyklema 1991), bacterial adhesion (Ferreirós et al. 1989; Kiremitçi-Gümüşderelioğlu and Peşmen 1996) and crystallization (Busscher et al. 2004; Wu and Nancollas 1997).

Urothelium is extremely hydrophilic (Cornish et al. 1990; Parsons 2007). When two hydrophilic bodies are brought into contact, water or physiological fluid present at the interface forms a stable fluid barrier, which acts as a sliding plane between both surfaces (e.g. between the medical device and tissue) exhibiting excellent low frictional properties. Thus, high surface wettability and low friction are considered to be directly linked properties (Borutto et al. 1998). The frictional force of a medical device can be reduced by more than 80% by highly lubricous coatings or other surface modifications (Nagaoka and Akashi 1990; Uyama et al. 1991), but not all studies comparing wettability and friction of urological implants find a significant correlation (Jones et al. 2004; Kazmierska et al. 2008). Nevertheless, a clinical study showed that stents with hydrophilic coatings induce less hematuria and pain than uncoated ones (Stensballe et al. 2005).

Most manufacturers of DJ-stents claim a high surface wettability (hydrophilicity) of their product. All DJ-stents are sold in dry packages. Some manufactures advise to soak not only the guide wire but also the implant in normal saline prior to insertion. The need of high lubricity (i.e. hydrophilicity or wettability) of the stent’s guide wire (Torricelli et al. 2013) is obvious, and thus its activation by pre-soaking is common practice.

Stent friction within the ureter may cause abrasions of the urothelium. In particular in patients with inflammation-related swollen ureteral orifice and ureter, tumor-compression or benign ureteral stricture, the need of stent-inflicted trauma-minimizing stent insertion is evident. In this case, the stent’s surface properties play a major role in its handling and indwelling-performance. Like for the guide wire hydrophilicity can be a major feature to achieve that goal for stents, too. Complications may be prevented not only by working under strictly sterile conditions and adequate antibiotic regimens, but also by addressing surgical techniques and technical issues (Tenke et al. 2014).

All implants claiming hydrophilicity need to be wetted before insertion. That is common frequent users knowledge, but an random inquiry of 25 urologists working in referral centers revealed that increasing time-pressure necessitates them to place the stent directly into the ureter without any moistening.

## Methods

### Wettability measurements

To determine the surface wettability of the samples, optical contact angle measurements were performed using the sessile drop method with the OCA15 plus instrument (DataPhysics Instruments GmbH, Germany) equipped with an electronically controlled microsyringe and a CCD-video camera.

**Figure 1:**
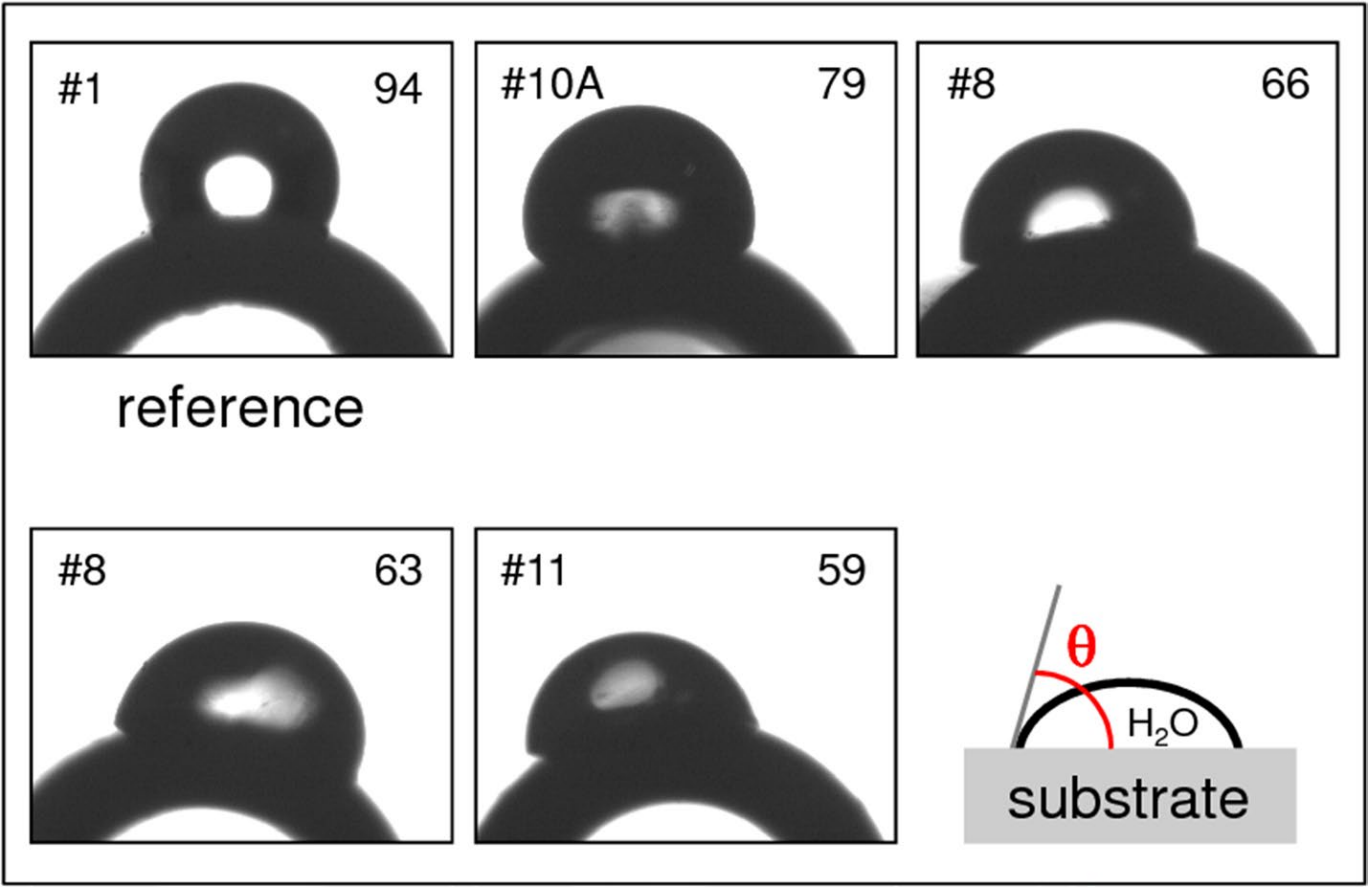
Examples of water contact angles θ observed on different surface types (labelled by *numbers* *in* *upper left corners*) of ureteral stents. *Numbers in upper right corners* indicate mean θ in degrees from *left*- and *right*-side measurement. The surface’s hydrophilicity/wettability increases with decreasing values of θ.

When using water as test liquid, the sessile drop technique is a routine method for the characterization of the wettability of a surface. The equilibrium water contact angle (WCA, θ) measured between the tangent to the drop’s profile and the tangent to the surface at the 3-phase contact point (i.e. the gas/liquid/solid intersection, Figure 1) is an index of the surface’s wettability: θ < 90° indicates a hydrophilic surface, in contrast, a surface with θ ≥ 90° is termed hydrophobic (Yuan and Lee 2013).

Sessile drops of 0.5 µl of deionized water (ROTIPURAN®, p.a., ACS, Carl Roth GmbH, Germany) were deposited on the dry stent samples (7 Charrière) and the contact angles were determined from photographs taken 5 s after drop deposition (Figure 1). For each measurement the baseline was manually defined according to the substrate curvature. The drop contour was calculated using an elliptic fit and θ was then determined from the slope of the contour line at the left and right 3-phase contact points (Ratner et al. 2004; Lam et al. 2001). The mean error of the WCA-measurements amounts to ±5% (ASTM 2001).

One reference surface and 11 different stent surface types from five different brand manufacturers were tested (Table 1). The investigated surfaces are considered to be particularly suitable for long-term indwelling as it can be assumed that their friction properties do not alter over time—in contrast to hydrogel coatings, which show (after mandatory soaking) very low friction during placement but can fall off within a short time after insertion thereby changing the stent’s sliding characteristics.

**Table 1:** Surface types of ureteral stents and quantities of specimens tested under dry and soaked conditions, respectively

Surface #	M	Type	Amount of samples	
			Dry	Soaked
1	1	Raw aliphatic PU	3	5
2	1	PU (“soft”)	1	2
3	2	Phosphorylcholine	2	3
4	1	PU (“strong”)	1	2
5	1	PU (“soft”)	1	2
6	1	PU (“strong”)	1	2
7	4	a-C:H (CF_4_)	3	5
8	4	a-C:H (N_2_)	3	4
9	4	a-C:H (1)	3	5
10A	4	a-C:H (2)	5	8
10B	5		2	3
11	3	“Soft”-PU	4	5

Surface type (e.g. “soft”, “strong”) according to available manufacturers (M) information. PU = proprietary polyurethane, a-C:H = hydrogenated amorphous carbon deposited from acetylene plasma (CF_4_ and N_2_ indicate additional process gases, 1 and 2 represent films deposited from pure plasma at different physical conditions). Surfaces #10A and #10B: same coating but different substrate materials.

*A* reference material (i.e. #1), *B* different proprietary PU.

As we would like to investigate potential effects of pre-soaking in general using quite different surface types, which however do not cover the whole spectrum of surface modifications on the market, the manufacturers are held anonymous. The information on stent materials and coatings included in this study is publicly available.

The reference consisted of uncoated aliphatic PU-tubes with a single batch number, supplied by the 1st stent manufacturer (surface #1). Surface types #2–6, #10B and #11 are from commercially available “hydrophilic” stents of clinical standard for long-term indication with dwelling times up to 12 months with a remaining shelf life of more than 6 months. The surfaces #2 and #4–6 are provided by the 1st manufacturer and nominally composed of the same proprietary PU, but differ in rigidity (“soft”–“strong”, according to the manufacturer) and color. #3 (2nd manufacturer) is provided with a phosphorylcholine functionalization and #11 (3rd manufacturer) is made of a different proprietary “soft” PU.

Surfaces #7–10A are experimental coatings of hydrogenated amorphous carbon (a-C:H), provided by the 4th manufacturer; #10B was supplied by manufacturer 5. a-C:H consists of amorphous carbon with a significant fraction of C–C sp^3^-bonds and H content in the range between 20 and 40 at.% (Casiraghi et al. 2005). They were deposited on the reference PU-tubes (#1) by radio-frequency plasma-enhanced chemical vapor deposition (RF-PECVD) from acetylene gas (C_2_H_2_) as described in (Kleinen et al. 2007). Surfaces #9 and #10A were deposited from pure C_2_H_2_-plasma, however, under different physical conditions (gas pressure, RF-power). To further adjust surface wettability, additional process gases were added: CF_4_ for #7 and N_2_ for #8. #10A and #10B are a-C:H coatings deposited by the same process on two different PU-materials.

In order to determine the influence of moistening on the stents’ surface wettability, contact angle determinations were performed twice, first with dry specimens, second with moistened specimens; the latter were thoroughly soaked in physiological NaCl-solution for 30 min to ensure a complete wetting. Before applying the sessile drop method, any surplus NaCl-solution was carefully removed with lint-free absorbent paper (KimtechScience® Kimwipes™, Kimberly-Clark, USA).

Each sample of a surface type was tested at three different positions to avoid effects of surface heterogeneities. Up to six samples of the same surface type were examined.

Mean relative humidity of ambient air during WCA-measurements was 53% (±10%); mean room temperature was 24°C (±1K).

### Statistical analysis

Correlations between two parameter sets were calculated according to the Pearson correlation coefficient (*r*).

The mean values of the datasets obtained from dry and wet WCA-measurements of each surface type were tested for being equal using a two-tailed paired student’s *t* test under the assumption of unequal variances. A *p* value ≤0.01 was considered to indicate a statistically significant difference.

## Results

No relevant linear correlation between WCA and room temperature as well as relative air humidity was observed (in both cases *r* = 0.24).

**Figure 2:**
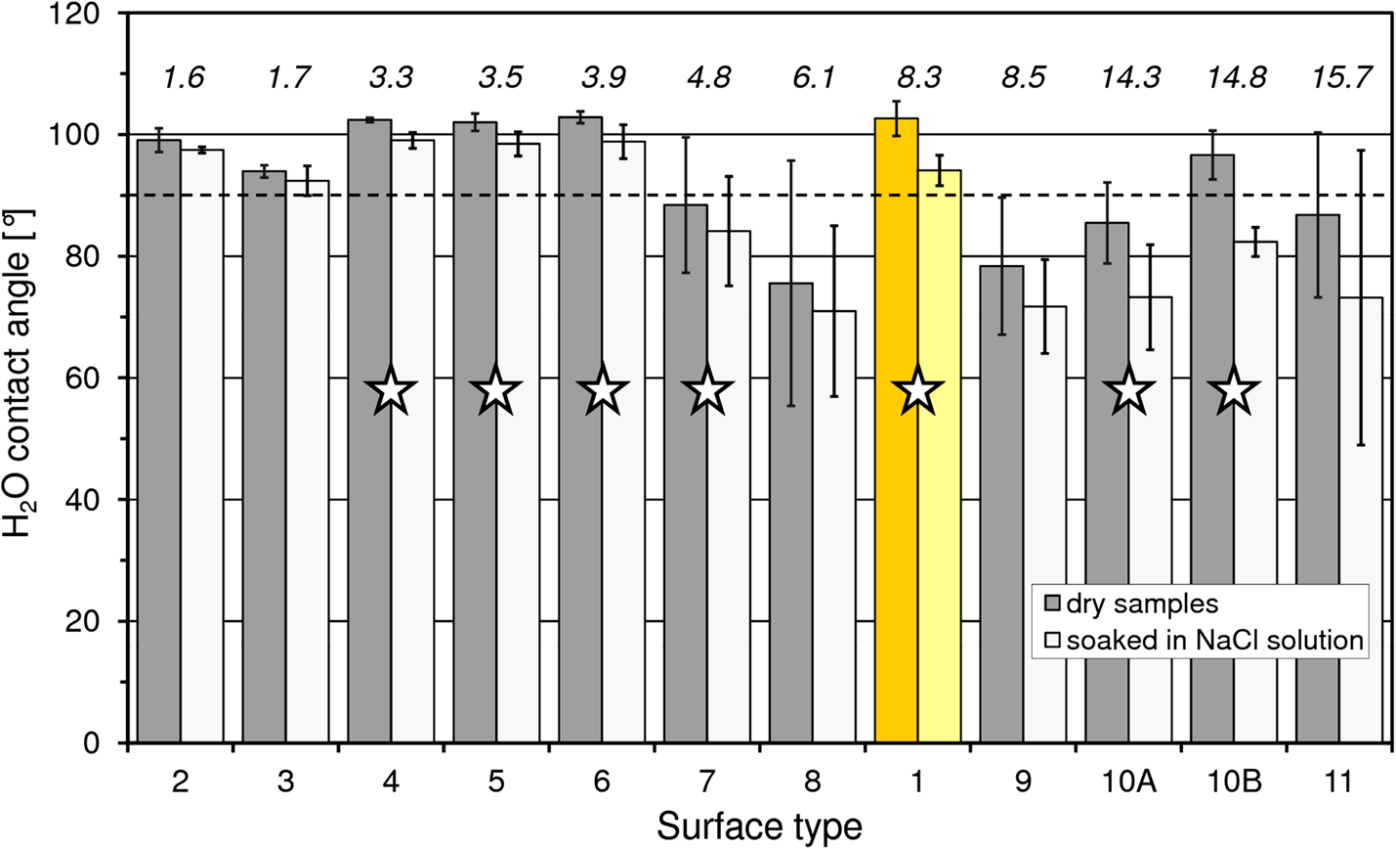
Results of optical WCA measurements (mean values ± SD) on various surfaces. #1: uncoated polyurethane reference; #2–6, #10B and #11: commercially available “hydrophilic” stents. #7–9, #10A: differently composed experimental coatings. Stent types are sorted according to increasing difference between “fresh” and “soaked” contact angle [*italic numbers* *on top of bars* in (%)]. *Star* mean value difference is statistically significant with *p* ≤ 0.01. *Dashed line* at 90° marks the hydrophobic–hydrophilic threshold (Yuan and Lee 2013).

Mean (median) WCAs on fresh and soaked stent surfaces ranged between 75° (67°) and 103° (103°), and between 71° (65°) and 99° (99°), respectively. In every case the WCAs on pre-wetted surfaces were lower than on fresh ones (Figure 2). Thus, any stent freshly taken out of its wrapping shows less hydrophilic properties compared to the same stent after soaking in saline. On average, the water contact angle decreases by 7%, the individual differences of the tested products differ considerably, from 2% (#2, #3 → PU or phosphorylcholine) to 16% (#11 → “soft”-PU). For 7/12 of the examined surface types, the decrease in contact angle is statistically significant with *p* ≤ 0.01.

Scattering of WCA-measurements on a particular specimen of a surface type is characterized by standard deviations between 0.3° (#1 → raw PU) and 8.3° (#8 → a-C:H(N_2_)) for fresh samples, and between 0.3° (#2 → PU) and 4.6° (#9 → a-C:H(1)) for pre-soaked samples.

The decrease in WCA due to soaking does not depend on the value of the initial contact angle determined from fresh stents (*r* = 0.36).

Surprisingly, the untreated reference stents (#1) made of hydrophobic polyurethane show similar contact angles as most of the commercial stents, which are claimed hydrophilic (#2, #4–6).

The different rigidity of the proprietary PU used in stent samples #2 and #4–6 does not significantly influence wettability or wettability increase after moistening. However, the wettability properties of these samples significantly differ from those of #11 made of a different proprietary PU.

## Discussion

Probability of micro-trauma to the urothelium and subsequent inflammatory response is reduced if the employed catheters and stents (as well as instruments) are characterized by low friction coefficients at the moment of placing and over the entire indwelling time (Stensballe et al. 2005; Torricelli et al. 2013). In particular for long-term implants, the surface should show resistance to mechanical abrasion in the hydrated state and stability against chemical aging.

High surface wettability is considered to be linked with low friction coefficients (Borutto et al. 1998). Thus, many urological implants are claimed being “especially hydrophilic”. However, all commercial stent types examined in this study show θ ≥ 90° directly after unwrapping; they are hydrophobic. Even after soaking in physiologic saline, only one of them shows θ < 90° (#10B); i.e. hydrophilic behavior.

More than half of the tested surface types show significantly higher wettability after being soaked in saline leading to the following conclusions:

1. In order to characterize the wettability properties of ureteral stents, measurements should be done on soaked samples.
2. To obtain maximum hydrophilicity at stent placement, stents should be soaked. Though technical advice is scarce in guidelines in general, the issue of wetting ureteral stents previous to insertion should be addressed. Similar assumptions may be made for any kind of urological equipment.

Most “out-the-bag” ready-to-use catheters for intermittent catheterization are either preconditioned with hydrophilic coating to be activated by breaking-off of an included pouch of sterile water that is released into the catheter package, or packed ready-to-use hydrogel. Such kind of packaging might be useful for urological implants indicated in tricky conditions, too, to obtain an appropriately moistened surface without the need to include a time-consuming soaking step in the tight schedule of a clinical intervention.

The same type of coating on different substrates does not necessarily result in the same surface wettability. This may be due to interactions between substrate and coating material during or after the coating process: The a-C:H surfaces #10A and #10B are deposited by the same process on different proprietary polyurethanes, whose respective compositions are not available. Thus, when developing new coatings, it should be tested, whether the properties adjusted on a “development surface” can be transferred to that of the final product.

On some stents WCAs show a surprisingly high scatter even on a single specimen. This can be explained by heterogeneities at microscopic and even nanoscopic scale at the surface due to variations in the production process, e.g. wear of the extruder head. Spatial variations in micro- or nano-roughness and surface chemistry as well as adhering contaminants can alter surface properties including wettability (Bico et al. 2008). Indeed, Wenzel’s wettability model predicts that a surface allowing water to make total contact at the solid–liquid interface will have its wetting properties magnified when the roughness is increased (Wenzel 1936; Kubiak et al. 2011). This means that a hydrophobic “perfectly smooth” surface (θ > 90°) will have its WCA increased when roughened, whereas a hydrophilic surface (θ < 90°) will have its WCA reduced upon roughening. Such a behavior has been confirmed for various polymers (Busscher et al. 1984).

## Conclusions

WCA-measurements on commercial and experimental urological stent surfaces have shown that directly after unwrapping most implants are actually hydrophobic contrary to the manufacturers’ claim of hydrophilicity. Nevertheless, hydrophilicity can be enhanced by proper submersing the stents in physiological saline. This not only may reduce frictional irritation and cell adhesion at the biomaterial–urothelial interface while indwelling but also eases stent insertion.

However, most uncoated (proprietary) PU products and the phosphorylcholine-coated samples still remain hydrophobic (i.e. θ > 90°). Only one uncoated PU product and the stents with amorphous carbon coatings get hydrophilic by soaking in saline. This paper does not intend to recommend a certain kind of stent for clinical interventions. This can only be done by clinical studies. Here, we concentrate on the general effect of pre-soaking of various stent materials on their wettability.

Obviously, stents are wetted during insertion since endourological procedures as ureteral stenting take place in an aqueous environment. Yet, during contact angle measurements of dry samples, changes in the respective drop contours could not be observed during 120 s after drop placement. Therefore just dipping the stent into saline before or bringing it into contact with urine just at the moment of placement seems to be too short to effectively reduce the contact angle, and thus to fully activate the products surface’s wettability.

Albeit the differences in contact angle between dry and soaked samples are statistically significant for more than half of the products, it should be clarified whether they are also of clinical relevance; i.e. whether soaked stents offer better handling for the clinician or reduced pain or lower incidence of urothelium injury for the patient as expected from the considerations in the introduction section. This is not in the scope of the present work and shall be the topic of forthcoming clinical studies.

Surface modification might be tailor-made to the particular types of microbial loads the patients suffer from, because different microbial strains adhere preferentially to surfaces with different characteristics, e.g. hydrophilicity (Ferreirós et al. 1989; Kiremitçi-Gümüşderelioğlu and Peşmen 1996). If patients are treated with stents designed to repel their particular microbial load, the probability of infection might be reduced.
